# Prevalence of Drug Resistance and Associated Mutations in a Population of HIV-1^+^ Puerto Ricans: 2006–2010

**DOI:** 10.1155/2012/934041

**Published:** 2012-04-24

**Authors:** Lycely del C. Sepúlveda-Torres, Alexandra De La Rosa, Luz Cumba, Nawal Boukli, Eddy Ríos-Olivares, Luis A. Cubano

**Affiliations:** ^1^Department of Microbiology and Immunology, Universidad Central del Caribe, P.O. Box 60327, Bayamón, PR 00960-6032, USA; ^2^School of Science and Technology, Universidad Metropolitana, P.O. Box 21150, San Juan, PR 00928-1150, USA

## Abstract

This is a continuation of our efforts to maintain a record of the evolution of HIV-1 infection in Puerto Rico by monitoring the expression levels of antiretroviral drug-resistance-associated mutations. Samples from 2,500 patients from 2006–2010 were analyzed using the TruGene HIV-1 genotyping kit and the OpenGene DNA sequencing system. Results show that 58.8% of males and 65.3% of females had HIV-1 with resistance to at least one medication. The average number of HIV mutations was 6.0 in males and 6.1 in females. Statistically significant differences between men and women were recorded in the levels of HIV-1 expressed mutations and antiretroviral drug resistance. The most prevalent antiretroviral medication resistance shifted from zalcitabine to nevirapine and efavirenz in the five-year period. M184V and L63P were the dominant mutations for the reverse transcriptase and the protease genes, respectively, but an increase in the incidence of minority mutations was observed.

## 1. Introduction

Puerto Rico has one of the highest incidences of HIV infections among the United States and it's jurisdictions. The official data collected by the Puerto Rico Department of Health indicate that the number of persons living with HIV increased from 39,455 to 43,400 from 2006 to 2010 and that 5,027 new diagnoses were performed during the same period of time (1,460 in 2006, 1,016 in 2007, 975 in 2008, 844 in 2009, and 732 in 2010) [1]. A comprehensive analysis of the HIV population in Puerto Rico was performed by the US Centers for Disease Control and Prevention (CDC) using the data for 2006. In that particular year, 71% of the new diagnosed individuals were males. By age group, the greatest number of diagnoses of HIV infection occurred among those aged 30 to 39 years, followed by those aged 40 to 49 years. Among males, the most common mode of HIV transmission was injection-drug use (40%), followed by male-to-male sexual contact (30%). Among females, the most common mode of HIV transmission was high-risk heterosexual contact (73%), followed by injection-drug use (27%). The rate of diagnosis of HIV infection in Puerto Rico in 2006 was 45.0 per 100,000 population, twice the estimated US rate (22.8) and 1.5 times the estimated rate for Hispanics in the United States (29.4). The incidence rate among males in Puerto Rico (62.0) was 1.8 times the rate among US males (34.3) and 1.4 times the rate among US Hispanic males (43.1). The incidence rate among females in Puerto Rico (29.8) was 2.5 times the rate among US females (11.9) and 2.0 times the rate in US Hispanic females (14.4) [2, 3]. However, comparisons between the rates for Puerto Rico and the rates for Hispanics in the United States should consider differences in the two populations since Hispanics in the United States include people of diverse national origins whose behavioral characteristics might differ from Hispanics in Puerto Rico [4]. One major difference between the HIV epidemiology in Puerto Rico and the United States is that injection-drug use continues to be the most common mode of HIV transmission in Puerto Rico, whereas most new HIV infections in the United States are attributed to male-to-male sexual contact [5, 6]. This observation is in agreement with previous reports that highlight a greater prevalence of injection-drug use and high-risk health behaviors related to injection-drug use (e.g., frequency of injecting and sharing syringes and other drug paraphernalia) in Puerto Rico than in the United States [4, 7].

Further analysis of the 2008 US HIV surveillance data generated an estimated lifetime risk for HIV diagnosis for the residents of Puerto Rico at 2.1%. This is approximately 3.5 times greater than that for whites and, among racial/ethnic populations, was greater than all populations except Blacks/African Americans [8]. The aforementioned data reflect the disparate nature of the HIV epidemic among Latinos in general and Puerto Ricans in particular. The national HIV/AIDS strategy calls for increased focus on interventions for Hispanics/Latinos, such as culturally and linguistically appropriate interventions that include effective communication strategies, expansion of HIV testing and diagnosis, and improved access to prevention, care, and treatment services to reduce the number of new HIV infections. The goal is to lower the estimated life risk for HIV diagnosis and to reduce the disproportionate impact of HIV in the Hispanic/Latino population [9].

The introduction of highly active antiretroviral therapy (HAART) into clinical practice for the treatment of HIV has led to dramatic reductions in mortality and morbidity [10]. This is largely because antiretroviral combination therapy can suppress the plasma HIV viral load below detectable limits and cause gradual elevation in CD4 cell counts, resulting in improved immune status for responsive patients who are compliant with therapy [11]. The emergence of drug-resistant viruses remains the limiting factor in HIV-1 management, being a major cause of treatment failure, AIDS clinical progression, and death [12].

International guidelines focus on the importance of tailoring antiretroviral therapy to the individual patient, on the basis of HIV-1 genetic data, integrated with clinical, laboratory, and therapeutic information [13, 14]. HIV drug-resistance testing is an intricate part of the therapy customization process to avoid the prescription of antiretroviral drugs that are not effective against the viral variant present in the patient. Genetic testing relies on the sequencing of genes essential for viral replication and survival, like the reverse transcriptase (RT) and protease resistance (PR), to detect mutations that are known to confer drug resistance.

Our institution has been providing HIV-genome sequencing services in Puerto Rico since 2000. This study is part of our continuing effort to establish an HIV-1 resistance-monitoring system in Puerto Rico, and an extension of previous reports that examined the prevalence of HIV-1-resistant mutations in the island from 2000 to 2005 [15–17]. Significant differences were recorded for various viral mutations and drug resistance between men and women from 2006 to 2010. Changes in drug resistance trends are discussed.

## 2. Methods

### 2.1. Samples

The Immunoretrovirus Research Laboratory located at the Universidad Central del Caribe in Bayamón, Puerto Rico provided the HIV-1 genotyping results from patients referred to the facility by their primary care physicians. Whole blood from HIV-1-infected patients was collected in tubes containing ethylenediaminetetra-acetic acid (EDTA) as anticoagulant. Plasma was separated and stored at −80°C until RNA isolation. Samples were analyzed from 2006 (245 women, 411 men, and 51 anonymous), 2007 (206 women, 384 men, and 18 anonymous), 2008 (245 women, 445 men, and 16 anonymous), 2009 (89 women, 224 men, and 27 anonymous) and 2010 (36 women, 54 men, and 49 anonymous).

Sex was the only demographic information disclosed by most patients. No clinical data was available to describe the relation between the genotyped samples and the newly diagnosed individual or the proportion of drug-naïve to drug-treated patients. A quality control system based on unique patient identification numbers was used to ensure that duplicate samples were eliminated from the dataset. Our samples represent approximately 6% of the HIV-infected population in the island.

### 2.2. RNA Isolation

 HIV-1 viral RNA was extracted using the QIAGEN QIAamp Viral RNA Mini Kit (QIAGEN, Valencia CA) as previously described [16]. Briefly, 140 *μ*L plasma was added to 560 *μ*L of buffer AVL containing carrier RNA in a microcentrifuge tube. Following incubation at room temperature for 10 min, 560 *μ*L of ethanol was added and mixed. Two consecutive aliquots of 630 *μ*L were added to the QIAamp spin column and centrifuged at 8,000 revolutions/min^−1^ (rpm) for 1 min. The QIAamp spin column was transferred into a clean 2 mL collection tube followed by the addition of 500 *μ*L of Buffer AW1, a centrifugation step as previously described, 500 *μ*L of Buffer AW2, and another centrifuged at 14,000 rpm for 3 min. The QIAamp spin column was placed in a clean 1.5 mL microcentrifuge and incubated with 60 *μ*L of buffer AVE at room temperature for 1 min before the viral RNA was harvested by a final centrifugation step at 8,000 rpm for 1 min. The samples were stored at −80°C until ready for analysis.

### 2.3. Mutational Analysis and Drug Resistance Correlations

Viral mutations were determined by analyzing the RNA samples with the TruGene HIV-1 genotyping kit and the OpenGene DNA sequencing system using a proprietary interpretative algorithm developed by the manufacturer (Siemens Healthcare Diagnostics, Deerfield, IL).

### 2.4. Statistical Analysis

InStat 3 for Macintosh (GraphPad Software Inc., La Jolla, CA) was used to perform the analysis. Fisher's exact test was performed. Statistical significance was set at *P* ≤ 0.05.

## 3. Results

### 3.1. General Gender Distribution of Viral Mutations and Resistance to Antiretrovirals

 Of 2,500 patients studied over a five-year period, the TruGene HIV-1 genotyping kit detected viral strains harboring at least one mutation in 96.3% of the women (791 of 821) and 96.9% of the men (1,471 of 1,518). Resistance to at least one medication was observed in 65.3% of the women (536 of 821) and 58.8% of the men (893 of 1,518). An average of 6.1 mutations was recorded per women, while the corresponding result for men was slightly less (6.0). Resistance to an average of 5.0 and 4.8 medications was noted for females and males, respectively. Taking in consideration only the patients infected with mutant viruses, an average of 6.4 mutations per women and 6.2 mutations per men was recorded. Likewise, the average number of medication resistance events was 7.6 for the women and 8.1 for the men showing resistance to any medication.

### 3.2. Antiretroviral Drug Resistance as Determined by TruGene HIV-1 Genotyping

Table 1 compares the levels of HIV-1 resistance to antiretroviral drugs from 2006 to 2010. In 2006, the highest rates of resistance were observed for zalcitabine (ddC) (397 of 707, 56.2%), lamivudine (3TC) (347 of 707, 49.1%), and atazanavir (ATV) (310 of 707, 43.8%). In 2007, the viral strains were most resistant to emtricitabine (FTC) (272 of 608, 44.7%), ATV (218 of 608, 35.9%), and nelfinavir (NFV) (204 of 608, 33.6%), and significant difference between men and women was observed for nevirapine (NVP) (*P* = 0.01) and efavirenz (EFV) (*P* = 0.001). The same three antiretrovirals attained the highest levels of resistance in the following two years with the most resistance events recorded for 3TC/FTC (219 of 706, 31.0% for 2008 and 95 of 340, 27.9% for 2009), followed by NVP (180 of 706, 25.5% in 2008 and 74 of 340, 21.8% in 2009) and EFV (175 of 706, 24.8% in 2008 and 71 of 340, 20.9% in 2009). In the case of 2010, NVP and EFV scored the highest level of resistance at 25 of 139 (18.0%) each, followed by saquinavir/ritonavir (SQV/r) at 21 of 139 (15.1%). Significant difference among genders was recorded for 3TC/FTC (*P* = 0.03) for the last year of the study.

### 3.3. Reverse Transcriptase Resistance-Associated Mutations

 The ten most prevalent RT resistance-associated mutations from 2006 to 2010 are reported in Table 2. M184V was the dominant mutation for all the years included in this study with frequencies of (322 of 707, 45.5%), (253 of 608, 41.6%), (206 of 706, 29.2%), (88 of 340, 25.9%), and (14 of 139, 10.1%), respectively. M41L was the second most common mutation for 2006 and the third most common mutation for 2007, 2008, and 2010 with prevalences of (157 of 707, 22.2%), (106 of 608, 17.4%), (79 of 706, 11.2%), and (11 of 139, 7.9%) for these years. K103N was also among the most important mutations since it was the third highest mutation for 2006 and remained in second place for the duration of the study with incidences of (152 of 707, 21.5%), (114 of 608, 18.8%), (143 of 706, 20.3%), (58 of 340, 17.1%), and (12 of 139, 8.6%) during the five-year period. V118I was the third highest mutation for 2009 with an occurrence of 38 of 340 (11.2%). Statistically significant differences between men and women were observed for K70R (*P* = 0.04) and K219Q (*P* = 0.03) in 2006, K103N (*P* = 0.02) in 2007, K219E (*P* = 0.03) in 2009 (data not shown), and V118I (*P* = 0.03) in 2010.

### 3.4. Protease Resistance-Associated Mutations

As shown in Table 3, the PR-associated mutations with the highest degree of expression from 2006 to 2010 was L63P with frequencies of (525 of 707, 74.3%), (429 of 608, 70.6%), (494 of 706, 70.0%), (238 of 340, 70.0%), and (93 of 139, 66.9%), respectively. M36I was the second most common mutation for 2006 (164 of 707, 23.2%), while this position was occupied by V77I for the reminder of the study with prevalences of (201 of 608, 33.1%), (222 of 706, 31.4%), (125 of 340, 36.8%), and (45 of 139, 32.4%). I13V was the third highest mutation for 2006, 2007, and 2009 and was recorded in (155 of 707, 21.9%), (190 of 608, 31.3%), and (109 of 340, 32.1%) of the specimens, respectively. Likewise, the third place corresponded to I62V in 2008 (211 of 706, 29.9%) and 2010 (37 of 139, 26.6%). Statistically significant differences between men and women were recorded for A71V (*P* = 0.05) and L90M (*P* = 0.04) in 2006, and M36I (*P* = 0.005) in 2008.

## 4. Discussion

This study is an effort to establish an HIV-1 resistance-monitoring system in Puerto Rico and a continuation of the articles published in 2002, 2008, and 2010 that examined the prevalence of HIV-1 mutations and antiretroviral resistance in the island from 2000 to 2005 [15–17]. As highlighted in our previous reports, statistically significant differences between genders were observed for both antiretroviral resistance levels and mutation incidences. It is of interest to note that some of the gender differences detected in this study were also observed in previous years. In the case of antiretroviral drug resistance, gender differences were recorded for nevirapine in 2004 and 2007. The statistically significant results for the reverse transcriptase mutation K70R have been noted for 2003 and 2006, while gender differences for K103N were observed in 2005 and 2007. Similarly, the protease resistance mutation L90M showed a divergent result between men and women in 2003, 2005, and 2006. Even though it is premature to draw conclusions based on the recurrence of these events, gender difference patterns may emerge once data from subsequent years are made available for analysis.

The results also provide a glimpse on the evolving nature of the HIV-1 viral strains circulating in Puerto Rico. As illustrated in Table 1, resistance to zalcitabine and lamivudine dominated the drug resistance landscape in 2006, following the trend observed in the previous years [16, 17]. Resistance to emtricitabine/lamivudine and nevirapine was more common in the intermediate years, followed by a codominance of nevirapine and efavirenz in the last year of the study. There is a dramatic difference in the range of antiretroviral drug resistance observed from 2006 to 2010. In the first year of the study, there was a difference of approximately 56 percentual points between the most abundant and the least common antiretroviral resistance levels, and the gap closed in the subsequent years until a difference of only 15 percentual points was obtained for 2010.

The reverse transcriptase mutation results indicate that M184V continues to be the most common mutation surveyed in our studies. This is consistent with reports based on large-scale HIV-1 genotypic analyses that identify M184V/I as the most prevalent nucleoside and nucleotide-analog-RT-inhibitor- (NRTI-) resistance mutations that overcome the effects of drugs like emtricitabine and lamivudine [18, 19]. M41L, the second most common mutation for 2006 and the third most common mutation for 2007, 2008, and 2010, is also a dominant NRTI-resistance mutation identified in the same studies. K103N, the third most prevalent mutation for 2006 and the second most common mutation from 2007 to 2010, is frequently observed among nonnucleoside-RT-inhibitor-(NNRTI-) resistance mutations that neutralize efavirenz and nevirapine [18, 20] and has been identified as a prevalent mutation in other retrospective studies [21].

As in previous years, the most prevalent protease resistance mutation corresponds to L63, a position that shows a great variation of amino acid substitutions contributing to antiretroviral drug resistance [22, 23]. M36I, a mutation that allows faster *in vitro* HIV-1 replication regardless of the presence of protease inhibitors, was the second most common mutation for 2006 [24]. V77I, a variant that has been identified as an important emerging substitution in HIV epidemiology studies, was the second most common mutation from 2007 to 2010 [25, 26]. The third position was occupied by I13V in 2006, 2007, and 2009, while this place corresponded to I62V in 2008 and 2010. These mutations have been associated with poor virological response to antiretroviral treatments [27–29].

The tables show a descending trend for most of the mutations, accompanied by an increase in the incidence of minority mutations. This observation is more evident in Table 3 where mutations that did not appear on the top ten mutations in previous years started to dominate the results landscape by the end of the study period. For example, I15V, a mutation highly prevalent in Chinese HIV-1^+^ patients who are drug users [30], appeared for the first time in 2007 with a prevalence of 9.5% (data not shown), doubled in 2008, and remained as one of the most common mutations for 2009 and 2010. This observation may be validated once the data corresponding to the subsequent years are included in the analysis. More information is needed before an ascending tendency of minority mutations can be established with certainty.

Although deaths of persons with HIV/AIDS reported to the national HIV/AIDS surveillance system and US Vital Statistics have followed similar patterns across most demographic and behavioral strata, including gender, age, geographic distribution, and race/ethnicity, substantial variation exists in the percentages of decline among different subgroups [31]. Members of minority racial/ethnic groups have a higher propensity to discontinue antiretroviral (ARV) therapy, experience more virologic failure, and show elevated morbidity [32–34]. In the particular case of Hispanics, this ethnic group has been identified as one of the socioeconomic disparities associated with suboptimal HIV care, including delayed HIV diagnosis and treatment, early discontinuation of therapy, higher ratios of progression of disease, and death [35–38]. Gender disparities have also been observed in the United States, and minority women are increasingly disadvantaged [39, 40]. Poverty, low health literacy, lack of family support, limited access to transportation, patient-provider issues, the organizational infrastructure of the health care facility visited, and the perceived HIV stigma within their communities are some of the issues associated with the inequality [41, 42]. The aforementioned problems, along with the inclusion of gender-specific issues into management strategies for HIV-infected women, including preconception and reproductive counseling, should be taken into consideration while developing health plans for these patients [43, 44]. Research targeted towards the reduction of HIV-related disparities, including the understanding of HIV/AIDS prevalence in women and minority groups, the detection of emerging incidence trajectories in these groups, and obtaining more information on how HIV infection as a chronic disease affects these individuals and their communities remains a high priority for the National Institutes of Health Trans-NIH AIDS research initiatives [45].

In the particular case of the race/ethnicity versus drug-resistance correlation, studies reporting HIV drug-resistance rates between races are contradictory since race is accounted for differences in some publications [46], while others noticed no differences attributable to race [47, 48]. In the case of ARV therapy tolerance, race has been correlated with alterations in metabolic and anthropometric measures where Latinos experienced the most unfavorable changes [49]. Minorities are also at higher risks of experiencing specific adverse events but not in the overall adverse event rates, all-cause mortality, or rates of toxicity-related treatment discontinuations [50]. A recent analysis of the data collected in ten studies found that the risk for virologic failure among patients with similar variant loads was higher among black and Hispanic patients. This relationship persisted, even after adjustment for differences in adherence rates, suggesting that socioeconomic factors, differences in levels of drug or alcohol abuse, or perhaps race-specific polymorphisms in the cytochrome P450 system may play an important role in this difference [51].

Gender differences in ARV treatment outcomes and drug-resistance mutations are also of interest. Several studies from developed countries have not identified gender as a predictor for primary drug resistance [52–54], while others report that mutation prevalence is higher in males [46]. In a recent study, females showed 2-fold odds of having virological failure compared with males at one year after genotype resistance testing, independent of race or a history of optimal treatment [55].

Treatments for HIV-infected women are usually based on efficacy and tolerability studies conducted in men since women are typically underrepresented in ARV treatment clinical trials [56, 57], and many female participants withdraw their consent prior to the conclusion of the study [58, 59]. Similar pharmacokinetics, treatment responses, and outcomes are recorded for men and women [60–62], but females are more susceptible to ARV treatment delay [63, 64], higher drug exposure due to lower weights [65], and physiological and metabolic differences affecting drug absorbance, toxicity, and retention [57, 66]. Malaise symptoms like rash, peripheral neuropathy, fatigue, weight loss, and feelings of vertigo/dizziness are frequently reported by women [67]. The aforementioned reasons, along with the psychosocial factors affecting treatment compliance [67, 68], account for a higher rate of treatment changes and poor adherence among women living with HIV.

Current evidence demonstrates that HIV ARV drug-resistance in resource-limited sites has neither emerged nor been transmitted to the degree that had initially been feared. However, due to a lack of standardized methodologies, HIV ARV resistance data from resource-limited sites can be difficult to interpret and may not provide the programmatic evidence necessary for public health action [69]. As predicted, the use of ARV drugs in resource-rich regions has exerted an increased evolutionary pressure on the virus, leading to a higher prevalence of antiretroviral drug resistant variants even among treatment-naïve individuals. In the particular case of the United States, a cohort study conducted in New York city found that the prevalence of overall transmitted resistance changed from 13.2% to 24.1% during the periods of 1995 to 1998 and 2003 to 2004 [70], and other studies reported an overall prevalence of transmitted drug resistance between 5 and 20% [71, 72]. Even though no specific drug resistance data has been published for Puerto Rico, resistant rates may be similar between Puerto Rico and the continental United States due to the observed shared infection pattern maintained by the high level of travel between the two jurisdictions.

Our study is limited by the lack of demographic and behavioral data that would allow the performance of multivariate statistical analyses that could explain the driving forces behind the reported statistically significant differences. Likewise, clinically relevant comparisons between drug-naïve and drug-experienced patients cannot be performed because the patients included in the study did not disclose their clinical history. TruGene was designed to detect HIV-1 subtype B, and even though some reports indicate that it can perform well on diverse HIV-1 subtypes [73], we do not know if the increase in prevalence of minority mutations is affected by the introduction on nonsubtype B variants in the studied population. Furthermore, the assay detects mutations in the coding regions of the RT and protease genes, but it was not designed to identify mutations in other regions of the viral genome that could contribute to ARV therapy resistance.

According to the manufacturer, the rules for the TruGene HIV-1 gentotyping assay are developed from the knowledge of a world-renowned panel of HIV experts that meet annually to review the latest HIV-1 clinical and research data. The resistance effects of mutations identified in the HIV-1 sample by the OpenGene DNA sequencing system are the culmination of a proprietary interpretative algorithm that considers published and nonpublished data, the recommendations of the annual expert review panel as well as alternative interpretative algorithms. Six TruGene guideline rules were released during the study period (version 10 in January of 2006, version 11 in September of 2006, version 12 in February of 2007, version 13 in November of 2007, version 14 in February of 2009, and version 15 in December of 2009) and present a major technical limitation. Since privacy issues impede the electronic storage of patient data, previously analyzed samples cannot be harmonized by being reanalyzed using the latest software version that may detect formerly unknown resistance patterns. Therefore, the periodic actualization of the software to reflect the current state of knowledge of mutation-resistance correlations can introduce artifacts that are beyond the control of the investigators. For example, Table 1 shows a shift in antiretroviral drug resistance from lamivudine in 2006 to emtricitabine in 2007 to lamivudine/emtricitabine for the rest of the study. Since both drugs are associated with the same major mutations [18], it is possible that the change in the mutation-resistance correlation is directly related to software updates made by the manufacturer. Therefore, it is recommended to emphasize the results reported for the actual mutation rates because the outcome will remain the same, even if the mutation's correlation to a specific drug changes in the future to accommodate the evolving nature of our understanding of the HIV antiretroviral drug resistance field.

Like many commercial screening platforms, TruGene is also affected by its inability to detect the presence of minority viral variants that can rapidly grow under drug selection pressure and can contribute to treatment failure [74–77]. This outcome may change once new high-throughput sequencing methods with the ability to quantify resistant variants, providing both proportional and absolute numbers of sequencing reads with a mutation, are readily available for clinical use. For example, ultradeep sequencing, a promising emerging technology, has been used to interpret ARV resistance in clinical trials [78, 79], and the ability of clinical laboratories to perform the method was examined in an international collaborative study [80]. On the other hand, before these new sensitive resistance technologies can be used to improve the clinical utility of viral genotyping in a cost-effective manner, important issues like a better definition of the level of sensitivity required to detect drug-resistant variants, the effects different variants have on treatment response, and the requirement for genotypic assays to provide information on resistance mutation linkage must be addressed by the scientific community [81, 82].

Aside from the aforementioned limitations, the fact that gender differences in HIV mutations and ARV drug resistance in Puerto Rican patients have been noted in the past ten years could point out possible differences in ARV treatment efficiency for this particular population. Our data could be of value for prospective cohort studies designed to study these differences in more detail, with the goal of establishing HIV mutation and resistance models tailored to the needs of Hispanics in general and Puerto Ricans in particular.

## 5. Conclusions

As observed in previous reports of HIV-1 mutation trends and antiretroviral resistance for Puerto Rican patients, statistically significant differences between genders were observed for both antiretroviral drug resistance levels and mutation incidences in the reverse transcriptase and protease genes. The most abundant antiretroviral drug resistance levels shifted from zalcitabine to nevirapine and efavirenz, and the gap between the most prevalent and the least common antiretroviral drug resistance counts closed significantly. Even though M184V and L63P continue to be the most prevalent mutations for the reverse transcriptase and the protease genes, respectively, a descending trend was observed for most of the mutations. An increase in the incidence of minority mutations was detected, suggesting that less common HIV-1 variants circulating in Puerto Rico may grow rapidly in subsequent years. This observation may be validated once the corresponding data are included in the analysis. More information is needed before an ascending tendency of minority mutations can be established with certainty.

## Figures and Tables

**Table 1:** Rates of HIV-1 resistance to antiretroviral drugs, Puerto Rico 2006–2010.

	2006 (number of samples) % of resistant isolates			2007 (number of samples) % of resistant isolates			2008 (number of samples) % of resistant isolates			2009 (number of samples) % of resistant isolates			2010 (number of samples) % of resistant isolates		

Drug*	Men (411)	Women (245)	Total** (707)	Men (384)	Women (206)	Total (608)	Men (445)	Women (245)	Total (706)	Men (224)	Women (89)	Total (340)	Men (54)	Women (36)	Total (139)
NNRTIs															

DLV	33.3	33.1	33.2	9.4	10.2	9.7	0	0	0	0	0	0	0	0	0
EFV	30.4	29.4	30.0	24.0	36.9	28.5***	23.8	26.9	24.8	21.9	21.3	20.9	14.8	27.8	18.0
ETR	0	0	0	0	0	0	0	0	0	0	0	0	1.9	11.1	6.5
NVP	31.1	29.8	30.6	26.0	36.4	29.6***	24.5	27.8	25.5	23.2	21.3	21.8	14.8	27.8	18.0

NRTIs															

3TC	48.4	51.8	49.1	0	0	0	0	0	0	0	0	0	0	0	0
3TC/FTC	0	0	0	0	0	0	28.8	35.5	31.0	25.9	34.8	27.9	7.4	25	12.2***
ABC	36.5	34.3	35.1	28.6	26.7	27.5	14.8	16.7	15.3	11.2	12.4	11.8	5.6	16.7	8.6
AZT	36.3	35.1	35.2	33.1	30.1	31.3	22.5	22.9	22.5	12.1	18.0	13.5	9.3	19.4	12.2
d4T	39.4	36.3	37.6	34.1	30.1	32.1	22.5	23.3	22.7	12.1	18.0	13.5	11.1	19.4	12.9
ddC	56.0	59.2	56.2	19.3	15.5	17.6	0	0	0	0	0	0	0	0	0
DDI	27.7	30.2	28.4	26.0	27.2	26.0	14.2	16.7	15.0	9.8	11.2	10.6	5.6	16.7	9.4
FTC	0	0	0	45.8	45.6	44.7	0	0	0	0	0	0	0	0	0
TDF	24.8	25.7	24.9	24.7	26.2	24.8	13.5	15.1	14.0	10.7	10.1	11.5	5.6	16.7	9.4

PIs															

APV	29.2	26.9	28.1	0	0	0	0	0	0	0	0	0	0	0	0
APV/FPV	0	0	0	28.1	22.3	25.7	14.6	17.1	15.6	14.7	15.7	15.0	9.3	13.9	11.5
APV/r or FPV/r	20.2	17.6	19.0	0	0	0	13.3	16.3	14.4	14.3	14.6	14.4	9.3	13.9	11.5
ATV	45.3	42.4	43.8	38.8	31.6	35.9	22.2	26.9	23.9	16.1	18.0	17.1	9.3	11.1	10.8
ATV/r	0	0	0	18.5	18.9	18.3	17.5	18.4	18.0	14.3	15.7	15.0	7.4	11.1	10.1
DRV/r	0	0	0	6.8	6.8	6.7	6.1	7.8	6.8	4.5	4.5	4.4	1.9	8.3	3.6
FPV/r	0	0	0	27.1	20.9	24.3	0	0	0	0	0	0	0	0	0
IDV	36.0	33.5	34.9	34.4	27.2	31.4	18.9	23.3	20.4	17.0	21.3	18.5	7.4	11.1	8.6
IDV/r	25.3	25.7	25.3	31.5	27.2	29.6	16.6	20.0	17.8	14.7	14.6	15.3	5.6	11.1	7.2
LPV/r	26.0	21.2	23.5	21.9	18.0	20.2	12.6	16.3	14.0	12.1	10.1	11.8	0	8.3	2.9
NFV	42.3	39.2	40.5	35.7	30.6	33.6	21.1	25.7	22.5	16.1	21.3	17.9	7.4	11.1	9.4
RTV	33.8	31.4	32.8	12.2	7.8	10.7	0	0	0	0	0	0	0	0	0
SQV	35.0	30.2	33.0	11.7	7.8	10.4	0	0	0	0	0	0	0	0	0
SQV/r	24.3	23.3	23.6	29.7	24.8	27.6	17.1	20.0	18.0	13.8	12.4	14.4	14.8	19.4	15.1
TPV/r	8.3	8.6	7.9	14.1	12.6	13.2	10.3	10.2	10.5	7.1	5.6	7.1	3.7	8.3	6.5

*Drug category abbreviations: NNRTIs—nonnucleoside analog reverse transcriptase inhibitors, NRTIs—nucleoside and nucleotide analog reverse transcriptase inhibitors, and PIs—protease inhibitors.

Drug name abbreviations: ABC—abacavir, APV—amprenavir, ATV—atazanavir, AZT—zidovudine, ddC—zalcitabine, DDI—didanosine, DLV—delavirdine, DRV—darunavir, d4T—stavudine, EFV—efavirenz, ETR—etravirine, FPV—fosamprenavir, FTC—emtricitabine, IDV—indinavir, LPV—lopinavir, NFV—nelfinavir, NVP—nevirapine, RTV—ritonavir, SQV—saquinavir, TDF—tenofovir, TPV—tipranavir, 3TC—lamivudine, and /r—drug combined with Ritonavir.

**Includes the anonymous samples.

***Statistically significant difference observed between men and women.

**Table 2:** Frequency of the 10 most prevalent resistance mutations in the HIV-1 reverse transcriptase gene, Puerto Rico 2006–2010.

	2006 (number of samples) % of resistant isolates			2007 (number of samples) % of resistant isolates			2008 (number of samples) % of resistant isolates			2009 (number of samples) % of resistant isolates			2010 (number of samples) % of resistant isolates		

Mutation	Men (411)	Women (245)	Total* (707)	Men (384)	Women (206)	Total (608)	Men (445)	Women (245)	Total (706)	Men (224)	Women (89)	Total (340)	Men (54)	Women (36)	Total (139)
M184V	44.3	49.0	45.5	43.2	41.3	41.6	27.0	33.9	29.2	24.6	32.6	25.9	7.4	16.7	10.1
M41L	20.7	25.3	22.2	16.9	18.9	17.4	11.2	11.0	11.2	8.0	15.7	10.6	3.7	13.9	7.9
K103N	20.9	21.6	21.5	15.9	24.3	18.8**	18.7	23.3	20.3	17.0	20.2	17.1	3.7	13.9	8.6
T215Y	17.5	20.8	19.0	15.4	15.0	15.0	9.4	9.0	9.2	7.1	4.5	6.5	1.9	8.3	4.3
D67N	20.9	16.7	18.5	16.7	13.1	15.0	9.0	10.2	9.6	7.6	12.4	9.1	3.7	8.3	5.0
V118I	13.9	17.1	14.7	13.8	10.7	12.5	7.4	7.3	7.6	12.1	9.0	11.2	3.7	19.4	7.2**
K70R	17.0	11	14.1**	15.4	10.7	13.3	9.9	8.6	9.5	5.8	12.4	7.4			
L210W	10.0	13.1	10.9	9.1	8.7	8.9	6.3	6.9	6.5	4.5	3.4	4.4	1.9	11.1	3.6
K219Q	11.9	6.5	9.3**	9.9	6.8	8.6	6.1	5.3	5.7	5.4	1.1	3.8			
T215F	7.3	5.7	6.6				4.3	3.7	4.0				3.7	5.6	4.3
T69N				3.6	6.8	4.6				3.6	3.4	3.5			
G190A													5.6	5.6	4.3
K70R													3.7	5.6	3.6
E44D													0.0	8.3	3.6

*Includes the anonymous samples.

**Statistically significant difference observed between men and women.

**Table 3:** Frequency of the 10 most prevalent resistance mutations in the HIV-1 protease gene, Puerto Rico 2006–2010.

	2006 (number of samples) % of resistant isolates			2007 (number of samples) % of resistant isolates			2008 (number of samples) % of resistant isolates			2009 (number of samples) % of resistant isolates			2010 (number of samples) % of resistant isolates		

Mutation	Men (411)	Women (245)	Total* (707)	Men (384)	Women (206)	Total (608)	Men (445)	Women (245)	Total (706)	Men (224)	Women (89)	Total (340)	Men (54)	Women (36)	Total (139)
L63P**	75.7	75.1	74.3	71.9	68.0	70.6	72.8	65.7	70.0	70.5	69.7	70.0	64.8	66.7	66.9
M36I	20.2	26.5	23.2	27.1	25.2	26.0	18.0	27.4	21.7***	18.3	27.0	20.0	9.3	22.2	16.6
I13V	21.2	23.7	21.9	33.3	28.2	31.3	27.6	24.9	26.2	33.5	28.1	32.1	29.6	16.7	25.9
A71V	23.4	16.7	20.4***	18.0	14.1	16.8	9.9	12.7	11.3	9.4	9.0	10.0			
V82F	16.3	20.4	18.4												
M36L	17.5	18.8	17.5												
A71T	15.1	13.1	14.9				12.8	13.1	12.6	9.8	13.5	10.6	9.3	5.6	7.9
L10I	16.1	10.6	13.7	14.3	12.6	13.5	15.1	13.9	14.7	11.6	20.2	14.4			
D30N	11.0	14.3	11.6												
L90M	13.4	7.8	11.2***	17.5	14.1	16.3									
V77I				32.3	35.0	33.1	33.9	27.4	31.4	33.5	43.8	36.8	31.5	27.8	32.4
I62V				19.5	20.9	20.1	29.7	29.8	29.9	27.2	30.3	29.7	22.2	27.8	26.6
I93L				16.9	17.0	16.8	25.6	20.8	23.8	22.3	32.6	26.5	24.1	27.8	23.0
M46I				11.7	9.2	10.7									
I15V							17.8	20.8	18.8	21.4	20.2	21.2	20.4	13.9	15.8
D60E							11.2	11.8	11.3				13.0	13.9	13.0
L19I													11.1	5.6	10.8

*Includes the anonymous samples.

**Includes all TruGene outputs for L63P, L63P/T, and L63T.

***Statistically significant difference observed between men and women.
